# Commentary: Implications of causality in artificial intelligence. Why Causal AI is easier said than done

**DOI:** 10.3389/frai.2024.1488359

**Published:** 2025-01-22

**Authors:** Jean-Christophe Bélisle-Pipon

**Affiliations:** Faculty of Health Sciences, Simon Fraser University, Burnaby, BC, Canada

**Keywords:** causalAI, AI ethics, unintended consequence, scalability, ethical issue, social implication

## Introduction

Cavique's (2024) article, “*Implications of Causality in Artificial Intelligence*,” presents a compelling case for the importance of causalAI. By focusing on cause-and-effect relationships rather than mere correlations, causalAI offers a pathway to more transparent, fair, and reliable AI systems. Cavique argues that causalAI is the least criticized approach compared to *responsible AI, fair AI*, and *explainable AI*, largely due to its scientific rigor and potential to reduce biases. However, despite its promise, causalAI is not without challenges. This commentary aims to assess some of these limitations and potential criticisms of causalAI as presented by Cavique, arguing that while it holds substantial promise, its implementation and practical application may be more complex and fraught with difficulties than the author suggests.

## The complexity and accessibility of causalAI

One of the primary challenges with causalAI lies in its complexity. CausalAI requires a deep understanding of causal inference and advanced statistical techniques, making it less accessible to most AI developers (Cox, 2023). Unlike correlation-based methods, which are widely understood and now relatively easy to implement, causal models demand a high level of expertise. Arguably, only a select group of experts can effectively design, implement, and interpret these models. This complexity can create barriers to entry for many organizations and individuals who might want to engage in developing or using causalAI for benefiting from the transparency and fairness that causalAI promises. This could exacerbate existing disparities in AI literacy, capacitation, and epistemic justice, potentially leading to an increased form of AI elitism, where only those with advanced skills, knowledge, and wealth of resources can fully participate in or critique causalAI development. This situation could undermine the broader goal of making its benefits accessible to a wide audience.

## Data requirements and assumptions

Causal AI requires high-quality data that captures both correlations and context (Vallverdú, 2024). In practice, such data is often scarce or costly, posing challenges for establishing accurate causal relationships Additionally, even when data is available, it may be incomplete or biased in ways that could skew causal inferences. The assumptions underlying causal models also warrant critical examination. CausalAI models often assume that all relevant variables have been identified and correctly measured. However, in practice, unmeasured confounders—variables that influence both the cause and effect—can distort causal estimates, leading to incorrect conclusions and as Rawal et al. (2024) put it there is a lack of *ground truth* for validation. This reliance on potentially faulty assumptions could result in AI systems that, while appearing transparent and fair, are actually based on flawed reasoning.

## Scalability and generalization concerns

Scalability is a major challenge for causal AI, as building and validating models is complex and resource-intensive. These models often require tailored adjustments for new contexts, limiting their generalizability compared to correlation-based methods. This limitation could hinder the practical application of causalAI in scenarios where scalability and adaptability are key. Specificity required by causal models may limit their ability to generalize across different datasets or environments. While correlation-based models can often be applied broadly with minimal adjustments, causal models may need to be tailored to the particularities of each new situation. This lack of generalizability could make causalAI less appealing in settings where adaptability is needed.

## Interpretive challenges

CausalAI is lauded for its potential to improve fairness and transparency in AI systems, but these benefits are not guaranteed. The causal relationships identified by AI systems are not immune to the biases present in the underlying data. If the data reflects existing societal biases or power dynamics, the causal models derived from it may inadvertently reinforce these issues. Even when accurately identifying cause-and-effect relationships, they may perpetuate societal biases, potentially reinforcing inequities if not designed inclusively. Interpretation of causal models can be influenced by the subjective perspectives of those designing or using them (Mittelstadt et al., 2019)—especially if the design of CausalAI is not inclusive and transparent, allowing for the active participation of stakeholders. This subjectivity introduces another layer of potential bias, as different stakeholders may have different interpretations of what constitutes a fair or just causal relationship. Ensuring that causalAI models are both fair and transparent requires careful consideration of these ethical and interpretive challenges, which are not easily addressed through technical solutions alone (Bélisle-Pipon et al., 2021).

## Implementation and integration costs

The practical implementation of causalAI also raises significant concerns. Implementing causal AI often requires significant changes to workflows and data processes, leading to time and cost barriers. This can deter organizations, especially if benefits are not evident upfront. Furthermore, the transition to causalAI could disrupt existing AI practices and lead to resistance from stakeholders who are comfortable with current methods. The need for specialized knowledge and expertise to implement and maintain causal models may further exacerbate these challenges, making the adoption of causalAI more difficult in practice than in theory.

## Unintended consequences

Finally, the focus on causality may introduce increased risks of unintended (and undetected) consequences. Causal AI, if based on flawed models or data, can lead to unintended negative outcomes. Adjustments to causal variables might have unforeseen side effects, particularly in less-documented contexts or marginalized populations, leading to data-driven biases (Norori et al., 2021). These unintended consequences highlight the need for a cautious and nuanced approach to applying CausalAI in practice. Beyond that, CausalAI does not address the underlying issues of fairness, representation and power imbalance—because it's causal from a data and AI point of view does not mean it is true and representative of reality. So even a CausalAI that is capable of grasp cause-and-effect relationships will be wrong in relation to non- or under-documented realities. An important example of this is about rarer phenomena and especially marginalized populations, which will not be better represented by CausalAI, nor better understood or more fully taken into consideration.

## Discussion

While Cavique's advocacy for causalAI is well-founded, highlighting its potential to address critical issues in AI, significant challenges still accompany this paradigm. Further research into causalAI is not only desirable but essential, as shifting from mere correlations to examining the underlying “why” behind observed relationships offers a more robust, adaptable, and transparent path forward. Indeed, Pearl and Mackenzie's (2018) emphasis on capturing the mechanisms underlying data could help propel AI beyond its current capabilities. Nevertheless, the complexity of causal inference—requiring specialized expertise, high-quality datasets, and sophisticated interpretive frameworks—means that these methods pose logistical and practical barriers. Yet the long-term potential remains considerable, suggesting that such advances could fundamentally reshape our understanding and pursuit of “intelligence” in AI systems.

Still, “causal” does not equate to “trustworthy,” nor does it inherently ensure ethical adherence or public trust. As noted elsewhere, trustworthy AI—particularly within healthcare—necessitates coordinated efforts among developers, policymakers, and (clinical) institutions to uphold ethical standards, transparency, and accountability (Bélisle-Pipon, 2024). Even sophisticated causal models, when developed in isolation or influenced by deregulation pressures, risk producing misleading or inaccurate outputs in high-stakes domains where human welfare is paramount. Additionally, practical limitations—from data collection hurdles to interpretability challenges and bias amplification-must be addressed to ensure causalAI can truly serve the public good.

Furthermore, the concept of “AI ethics dumping” underscores how advanced causal methods may inadvertently shift ethical responsibilities onto communities with minimal control over AI (Bélisle-Pipon and Victor, 2024). Whether causal or correlation-based, many AI models embed normative assumptions without fully accounting for local realities, leaving those “on the ground” to manage ethical dilemmas with AI tools they did not help shape. This dynamic runs contrary to the goals of AI ethics and responsible innovation, as it risks perpetuating social and power imbalances. Incorporating community perspectives and stakeholder insights across every phase of causalAI—from inception and model design to overall governance, and particularly through co-reasoning approaches (Pacia et al., 2024)—helps mitigate these risks and to validate whether that ground truth that underlie causality is relevant and meaningful to them. Ultimately, embracing inclusive participatory methods and transparent accountability mechanisms is vital to ensuring that causalAI fulfills its promise as an answer to current correlation-based models and to further equitable AI benefits than an inadvertent driver of harm. Moving forward, addressing the complexities, data requirements, and ethical concerns outlined here will be crucial for unlocking causalAI's full potential and advancing beyond the inherent constraints of correlation-based models.

## References

[B1] Bélisle-Pipon J.-C. (2024). Why we need to be careful with LLMs in medicine. Front. Med. 11:1495582. 10.3389/fmed.2024.1495582 39697212 PMC11652181

[B2] Bélisle-Pipon J.-C. Couture V. Roy M.-C. Ganache I. Goetghebeur M. Cohen I. G. (2021). What makes artificial intelligence exceptional in health technology assessment? Front. Artif. Intell. 4:736697. 10.3389/frai.2021.736697 34796318 PMC8594317

[B3] Bélisle-Pipon J.-C. Victor G. (2024). Ethics dumping in artificial intelligence. Front. Artif. Intell. 7:1426761. 10.3389/frai.2024.1426761 39582547 PMC11582056

[B4] Cavique L. (2024). Implications of causality in artificial intelligence. Front. Artif. Intellig. 7:1439702. 10.3389/frai.2024.1439702 39233891 PMC11371780

[B5] Cox L. A. (2023). “Causally explainable decision recommendations using causal artificial intelligence,” in AI-ML for Decision and Risk Analysis: Challenges and Opportunities for Normative Decision Theory (Cham: Springer International Publishing), 273–316.

[B6] Mittelstadt B. Russell C. Wachter S. (2019). “Explaining explanations in AI,” in FAT* '19: Proceedings of the Conference on Fairness, Accountability, and Transparency, 279–288. 10.1145/3287560.3287574

[B7] Norori N. Hu Q. Aellen F. M. Faraci F. D. Tzovara A. (2021). Addressing bias in big data and AI for health care: a call for open science. Patterns 2:10. 10.1016/j.patter.2021.100347 34693373 PMC8515002

[B8] Pacia D. M. Ravitsky V. Hansen J. N. Lundberg E. Schulz W. Bélisle-Pipon J. C. (2024). Early AI lifecycle co-reasoning: ethics through integrated and diverse team science. Am. J. Bioethics 24, 86–88. 10.1080/15265161.2024.2377106 39226006

[B9] Pearl J. Mackenzie D. (2018). The Book of Why: the New Science of Cause and Effect. New York: Basic Books, 432.

[B10] Rawal A. Raglin A. Rawat D. B. Sadler B. M. McCoy J. (2024). Causality for trustworthy artificial intelligence: status, challenges and perspectives. ACM Comput. Surv. 10.1145/3665494

[B11] Vallverdú J. (2024). Causality for Artificial Intelligence: From a Philosophical Perspective. Cham: Springer Nature.

